# Deep learning segmentation of the cerebral ventricular system and brainstem for pediatric radiotherapy planning

**DOI:** 10.1016/j.phro.2026.101086

**Published:** 2026-09-19

**Authors:** Feline Heinzelmann, Fabian Hörst, Lukas Heine, Jana Fragemann, Moritz Rempe, Gijs Luijten, Sarah Peters, Julian Hlouschek, Jan Egger, Beate Timmermann, Jens Kleesiek, Christian Bäumer

**Affiliations:** a West German Proton Therapy Centre Essen (WPE), Essen, Germany; b TU Dortmund University, Department of Physics, Dortmund, Germany; c West German Cancer Center (WTZ), University Hospital Essen, Essen, Germany; d Institute for Artificial Intelligence in Medicine (IKIM), University Hospital Essen, Essen, Germany; e Center for Virtual and Extended Reality in Medicine (ZvRM), University Hospital Essen (AöR), Essen, Germany; f Clinic for Particle Therapy, University Hospital Essen, Essen, Germany; g German Cancer Consortium (DKTK), Essen, Germany; h University of Duisburg-Essen, Faculty of Medicine, Essen, Germany

**Keywords:** Deep learning segmentation, nnunet model, Ventricular system, Brainstem, Pediatric brain tumor, Radiotherapy planning

## Abstract

**Background and Purpose:**

Segmentation of the cerebral ventricular system (VS) is central to radiotherapy, both for whole-ventricular irradiation in intracranial germ cell tumors (GCTs) and protection of the periventricular region. Complex, variable VS anatomy makes manual delineation challenging and hampers auto-segmentation model development. We reported the evaluation and workflow integration of a deep-learning (DL) model for VS and brainstem segmentation on pediatric magnetic resonance imaging (MRI).

**Materials and methods:**

An nnU-Net was trained on MRIs from 76 unique brain tumor cases (*n* = 73/71 for T1/T2). The evaluation used two independent retrospective cohorts: (i) GCTs evaluated on planning computed tomography (CT) images after MRI-to-CT registration mapping (*n* = 13/14), and (ii) brain tumors evaluated on MRI (*n* = 14). Performance was assessed using the Dice similarity coefficient (DSC) and Hausdorff distances (HD) against manual contours. Prospective deployment in routine clinical planning assessed usability and workflow integration.

**Results:**

In the GCT-CT cohort, brainstem segmentations achieved median DSCs of >0.85 and HDs_95_ of ≤5.0/6.4 mm, while VS segmentations achieved DSCs of >0.70 and HDs_95_ of ≤5.3/4.3 mm, after rigid/deformable mapping to planning CT. In the MR cohort, performance on T1−/T2-weighted MRI reached DSCs of ≥0.90 for both structures, and HDs_95_ of ≤3.0/3.4 mm and ≤ 1.3/2.2 mm for the brainstem and VS, respectively. The DL-segmentation workflow reduced workflow time by >50% compared with manual contouring.

**Conclusions:**

Lower performance in the GCT-CT cohort likely reflects noisier CT-based references, interobserver variability, and uncorrected geometric/anatomical mismatch. The model's accuracy supports radiotherapy planning in pediatric brain tumors through risk-structure delineation and target-volume segmentation in GCTs.

## Highlights

• Automated ventricular system and brainstem segmentation developed for pediatric use.
• Quantitative and qualitative validation (Likert 4/5) in pediatric cohorts.
• Dice similarity scores ≥0.90, Hausdorff distances ≤3.4 mm for both organs on MRI.
• Contouring workflow time was reduced by ≈50%, despite high anatomical variability.

## Graphical abstract

**Figure:**
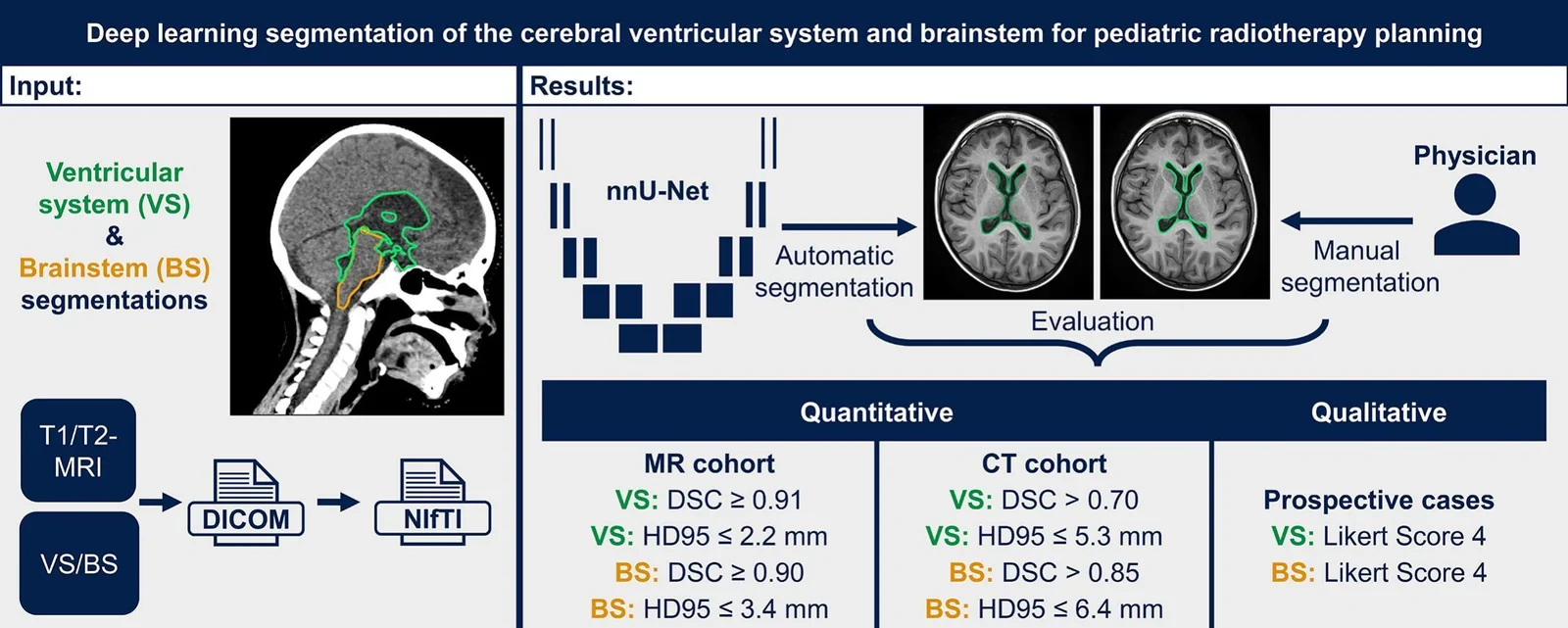

## 1 Introduction

Segmentation of the cerebral ventricular system (VS) is increasingly important in radiotherapy for both target-volume (TV) definition and organ-of-interest (OoI) assessment [1]. Traditionally, the VS has been a focus in radiotherapy planning for central nervous system (CNS) germ cell tumors (GCTs), particularly for pediatric patients. GCTs constitute a heterogeneous group of neoplasms, broadly classified as germinomatous and non-germinomatous germ cell tumors [2]. In general, patients with CNS GCTs should receive a combination of chemotherapy and RT, while surgery is usually limited to biopsy or complication management, given the deep and midline location of most tumors [2], [3], [4]. Whole-ventricular volume (WVV) irradiation in GCTs—especially those with a germinoma component—is critical [2], [5], [6], as underdosing of the TV may increase the risk of recurrence and compromise overall survival [7]. As this tumor entity primarily affect children and adolescents [8], whose higher vulnerability mandates careful aftercare, accurate delineation of the VS is essential to balance tumor control, late effects, and recurrence. 

In addition to the VS's role in TV definition for GCTs, recent studies have identified the periventricular region (PVR) —the 3–5 mm rim around the VS—as a radiosensitive OoI in RT, including proton beam therapy. The PVR's response to doses in the therapeutic regime is sometimes manifested as imaging changes. Therapy-induced damage to these structures has been associated with neurocognitive decline and other late adverse effects [9], [10], [11], [12], [13], [14], [15].

The VS is anatomically complex, with highly variable morphology and often poor visibility on computed tomography (CT). Reliable contouring therefore requires magnetic resonance imaging (MRI), whose superior soft-tissue contrast and cerebrospinal fluid (CSF)–parenchyma separation make it indispensable for VS and brainstem delineation [16], [17]. Nevertheless, routine practice still relies on labor-intensive manual contouring, driving the need for automated solutions. Deep learning (DL) auto-segmentation shows clear benefits in reducing workload and enhancing reproducibility in radiotherapy planning, but the deployment for the VS segmentation is constrained by scarce training/validation contours, largely due to cross-vendor and cross-sequence MRI heterogeneity and the PVR's recent designation as an OoI.

The present work describes the clinical validation and integration of an nnU-Net DL model for automated segmentation of the VS and brainstem on T1- and T2-weighted MR images (T1wIs/T2wIs) for pediatric radiotherapy patients, for whom no commercial, clinically validated automated segmentation is yet available.

## 2 Material and methods

### 2.1 Patient cohort

All included patients in the training and validation cohorts received proton beam therapy at the West German Proton Therapy Centre Essen, Germany. The legal guardians of the patients signed an informed consent form allowing the use of image data for retrospective studies. Treatment planning and contouring were performed within the treatment planning system RayStation© (RaySearch Laboratories, Stockholm, Sweden; version 2024 A SP1).

The DL model was trained on T1wIs and/or T2wIs with corresponding structure data from 76 pediatric patients (≤18 years) with malignant intracranial brain tumors of various entities, including only one imaging time point per patient (see Table 1). The patients were enrolled in the prospective registry study (KiProReg; German Clinical Trials Register: DRKS-ID: DRKS00005363).

**Table 1:** Patient demographics, image acquisition, and image details from the MRI data sets on which manual contouring for training or the automatic contouring for validation were performed first. For the CT validation cohort (ventricular system as a target), the CT acquisition and image details are additionally listed, since the evaluation was performed on the CT.

			**Median (Min, Max)/No.**		
	**Category**		**Training cohort**	**MRI validation cohort**	**CT validation cohort**
**Patient demographic**	**No. cases**		76	14	14
	**Age (y)**		9.3 (0.9, 17.4)	8.1 (1.2, 18.0)	13.7 (5.0, 17.3)
	**Gender**		31 female, 45 male	7 female, 7 male	4 female, 10 male
	**Entity**	**CNS GCT**	9	4	14
		**Medulloblastoma**	11	5	0
		**Ependymoma**	23	2	0
		**Glioma**	12	3	0
		**AT/RT**	7	0	0
		**Craniopharyngioma**	9	0	0
		**CPT**	2	0	0
		**RGNT**	1	0	0
		**Neuroblastoma**	1	0	0
		**Ganglioglioma**	1	0	0
**MR image details**		**Machine**^†^ **(model;** **software version;** **no. of T1/T2 sequences)**	**MRT-200**^††^ by Toshiba Medical Engineering Co. (V9.50*R249) **(T1:70; T2:68)**; **Avanto** by SIEMENS (syngo MR B19) **(T1:1; T2:1)**; **Aera** by SIEMENS (syngo MR E11) **(T1:1; T2:0)**; **Achieva** by Philips Medical Systems (3.2.3.2) **(T1:0; T2:1)**; **Intera** by Philips Medical Systems (12.6.1.4) **(T1:1; T2:0)**; **Ingenia** by Philips Medical Systems (5.1.7.2) **(T1:0; T2:1)**	**MRT-200**^††^ by Toshiba Medical Engineering Co. (V9.50*R249) **(T1:14; T2:14)**	**MRT-200**^††^ by Toshiba Medical Engineering Co. (V9.50*R249) **(T1:13; T2:14)**
	**T1**	**Sequence**	FFE3D4.5; *tfl3d1_ns; *tfl3d1_16ns	FFE3D4.5	FFE3D4.5
		**No. sequences**	73	14	13
		**No. transversal slices**	192 (25, 262)	192 (164, 232)	200 (178, 252)
		**Voxel size in x (mm)**	0.45 (0.34, 0.90)	0.36 (0.36, 0.50)	0.46 (0.36, 0.50)
		**Voxel size in y (mm)**	0.45 (0.34, 0.90)	0.36 (0.36, 0.50)	0.46 (0.36, 0.50)
		**Voxel size in z (mm)**	2.0 (1.0, 5.0)	2.0 (2.0, 2.0)	2.0 (2.0, 2.0)
		**Slice spacing (mm)**	1.0 (1.0, 6.0)	1.0 (1.0, 1.0)	1.0 (1.0, 1.0)
		**Image rows**	544 (256, 628)	576 (416, 576)	544 (496, 648)
		**Image columns**	544 (256, 576)	576 (416, 576)	544 (496, 576)
		**TE (ms)**	4.5 (2.9, 15.0)	4.5 (4.5, 4.5)	4.5 (4.5, 4.5)
		**TR (ms)**	10 (9, 1980)	10 (10,11)	11 (10, 11)
		**TI (ms)**	400 (400, 1100)	400 (400, 400)	400 (400, 400)
	**T2**	**Sequence**	FSE+13.3_nBW_slt; FSE+15_nBW_slt; *spcR3d1_87	FSE+13.3_nBW_slt; FSE+15_nBW_slt;	FSE+13.3_nBW_slt
		**No. sequences**	71	14	14
		**No. transversal slices**	63 (31,110)	67 (39, 100)	79 (45, 120)
		**Voxel size in x (mm)**	0.41 (0.30, 0.90)	0.38 (0.38, 0.49)	0.41 (0.38, 0.55)
		**Voxel size in y (mm)**	0.41 (0.30, 0.90)	0.38 (0.38, 0.49)	0.41 (0.38, 0.55)
		**Voxel size in z (mm)**	2.0 (0.8, 4.0)	2.0 (2.0, 2.0)	2.0 (2.0, 2.0)
		**Slice spacing (mm)**	2.2 (2.2, 4.6)	2.2 (2.2, 2.2)	2.2 (2.2, 2.2)
		**Image rows**	576 (256, 688)	576 (448, 608)	576 (452, 600)
		**Image columns**	512 (230, 640)	576 (448, 576)	512 (384, 576)
		**TE (ms)**	120 (80, 145)	120 (90, 120)	120 (120,120)
		**TR (ms)**	5200 (1200, 7800)	4900 (4050, 6300)	4900 (3700, 6300)
**CT image details of the CT validation cohort**		**Machine**			**Brilliance Big Bore** by Philips
		**Software**			2.3.5; 4.2 **(14)**
		**No. sequences**			14
		**No. transversal slices**			300 (260, 335)
		**Voxel size in x (mm)**			0.92 (0.68, 1.17)
		**Voxel size in y (mm)**			0.92 (0.68, 1.17)
		**Voxel size in z (mm)**			1.0 (1.0, 1.0)
		**Image rows**			512 (512, 512)
		**Image columns**			512 (512, 512)
		**Reconstruction diameter (mm)**			472.5 (350, 600)
		**Pitch**			0.56 (0.56, 0.56)
		**kVp (kV)**			120 (120, 120)
		**Tube current (mA)**			210 (150, 263)
		**Exposure time (ms)**			1333 (1330,1333)
		**Exposure (mAs)**			280 (200, 350)
		**CTDIvol (mGy)**			33.3 (23.8, 39.1)

**Abbreviations:** No., number; MRI, magnetic resonance imaging; CT, computed tomography; y, years; CNS, central nervous system; GCT, germ cell tumor; AT/RT, atypical teratoid rhabdoid tumor; CPT, choroid plexus tumor; RGNT; TE, echo time; TR, repetition time; TI, inversion time; kVp, peak kilovoltage; CTDIvol, CT dose index volume.

**Annotations:**^†^For each patient, the MRI scans were typically acquired alongside the planning CT (pCT) to guide radiotherapy planning. When reliable MRI from external institutions meeting the same quality criteria were available, repeated MRI acquisition in the frame of radiotherapy planning at West German Proton Therapy Centre was omitted. ^††^Toshiba Vantage Titan Large Bore 1.5 T, now operated by Canon Medical Systems.

### 2.2 Ventricular system and brainstem definition

The manual training contours for the VS are based on the protocol “Whole Ventricle Target Volume Atlas for Germ Cell Tumors (ACNS 1123)” for GCTs [18]. Brainstem segmentation was based on the German guideline “SIOP Ependymoma II”, which defines the upper limit of the brainstem as the upper aspect of the mesencephalon (below the third ventricle and the optic tract), and the lower limit as the inferior border of the foramen magnum [19].

### 2.3 Deep learning model architecture and training

The segmentation models were based on nnU-Net by Isensee et al. [20] (version 2, [21]), a widely adopted biomedical supervised segmentation framework. This framework offers a complete training and validation pipeline with automatic pre- and postprocessing of the input scan, as well as selecting network and training parameters. Despite the multi-channel capability of the nnU-Net, two separate single-sequence models—T1-only and T2-only—were trained to remain applicable when one sequence is unavailable. The framework performed randomized five-fold cross-validation to create patient-wise training and validation splits. Learning rate and loss function were fixed training parameters, while the network depth was selected by the dataset fingerprint [20]. Apart from the conversion from Digital Imaging and Communications in Medicine (DICOM) to Neuroimaging Informatics Technology Initiative (NIfTI) format and the nnU-Net's built-in normalization and resizing-to-target steps, no further preprocessing was applied. Based on the comparable performance of the 2D and 3D configurations (see Table S1), the two final models (accessible at https://github.com/TIO-IKIM/Ventricle-Segmentation-Model; see Supplementary material A) were implemented as 2D ensembles, each combining the five best checkpoints from the patient-wise five-fold cross-validation. The 2D models represent the more practical choice, given their substantially lower computational burden and shorter training and inference times, as well as the limited additional through-plane context obtainable from the thick-slice input images.

### 2.4 Deployment within the clinical system

Brainstem and VS contours generated by the DL model on T1wIs/T2wIs were stored as DICOM RT Structure Sets linked to the corresponding MRI series, subsequently imported into RS, and then mapped from MRI to the planning CT (pCT) using rigid or deformable registration (see the detailed workflow in Supplementary material B).

### 2.5 Retrospective pediatric GCT cohort with target contour evaluation on the pCT

Pediatric patients with CNS GCTs who received radiotherapy at our institute were eligible if a germinoma component was present and a WVV contour was available as the clinical TV. VS and brainstem contours on the pCT had to be approved by at least two board-certified radiation oncologists. Patients were excluded if they were older than 18 years at completion of proton beam therapy or if no same-day planning MRI (pMRI) was available. In patients with prior third ventriculostomy or evidence of, or high risk for, tumor extension into the prepontine cistern, the prepontine portion of the VS was retrospectively removed, even though the ACNS 1123 atlas mandates its inclusion in these cases [22]. A total of 14 patients were included (see Table 1). DL-generated structures were mapped from the respective pMRI to the pCT (see Section 2.4), thereby providing an evaluation of the segmentation workflow within CT-based treatment planning rather than constituting a purely independent validation cohort.

### 2.6 Retrospective pediatric brain tumor cohort with contour evaluation on the pMRI

Manual and DL-generated VS contours were evaluated on the same MRI, enabling direct comparison without the MRI–pCT registration and structure mapping required in the GCT-CT cohort. A physician and a physicist retrospectively delineated the VS and brainstem on T1wIs and T2wIs from 14 patients, followed by assessment by a senior radiation oncologist. Further cohort details are listed in Table 1.

Both cohorts described in 2.5, 2.6 were evaluated using the Dice similarity coefficient (DSC), the maximum and 95th-percentile Hausdorff distance (HD_max_/HD_95_) (see Supplementary material B).

### 2.7 Integration of the DL auto-contouring model into the clinical workflow

Clinical applicability beyond benchmark metrics was prospectively assessed in two CNS GCT cases. The workflow in Section 2.4 was applied, DL structures were conservatively refined with contour-enhancement tools in RayStation© for final clinical application, and qualitative performance was rated using a 5-point Likert scale and an extended version (see Tables S4 and S5).

## 3 Results

Overall, the models demonstrated favorable MRI-based segmentation performance across the two retrospective validation cohorts, as reflected by high median DSC values and low-to-moderate median HD_95_ values (Fig. 1, Fig. 2 and Tables S6–8). In the CT cohort evaluating the end-to-end workflow, brainstem segmentations achieved median DSCs of >0.85 and HDs_95_ of ≤5.0/6.4 mm, while VS segmentations achieved DSCs of >0.70 and HD_95_ of ≤5.3/4.3 mm, after rigid/deformable mapping to the pCT. In the MR cohort, performance on T1−/T2-weighted MRI reached DSCs of ≥0.90 for both structures, and HDs_95_ of ≤3.0/3.4 mm and ≤ 1.3/2.2 mm for the brainstem and VS, respectively.

**Fig. 1:**
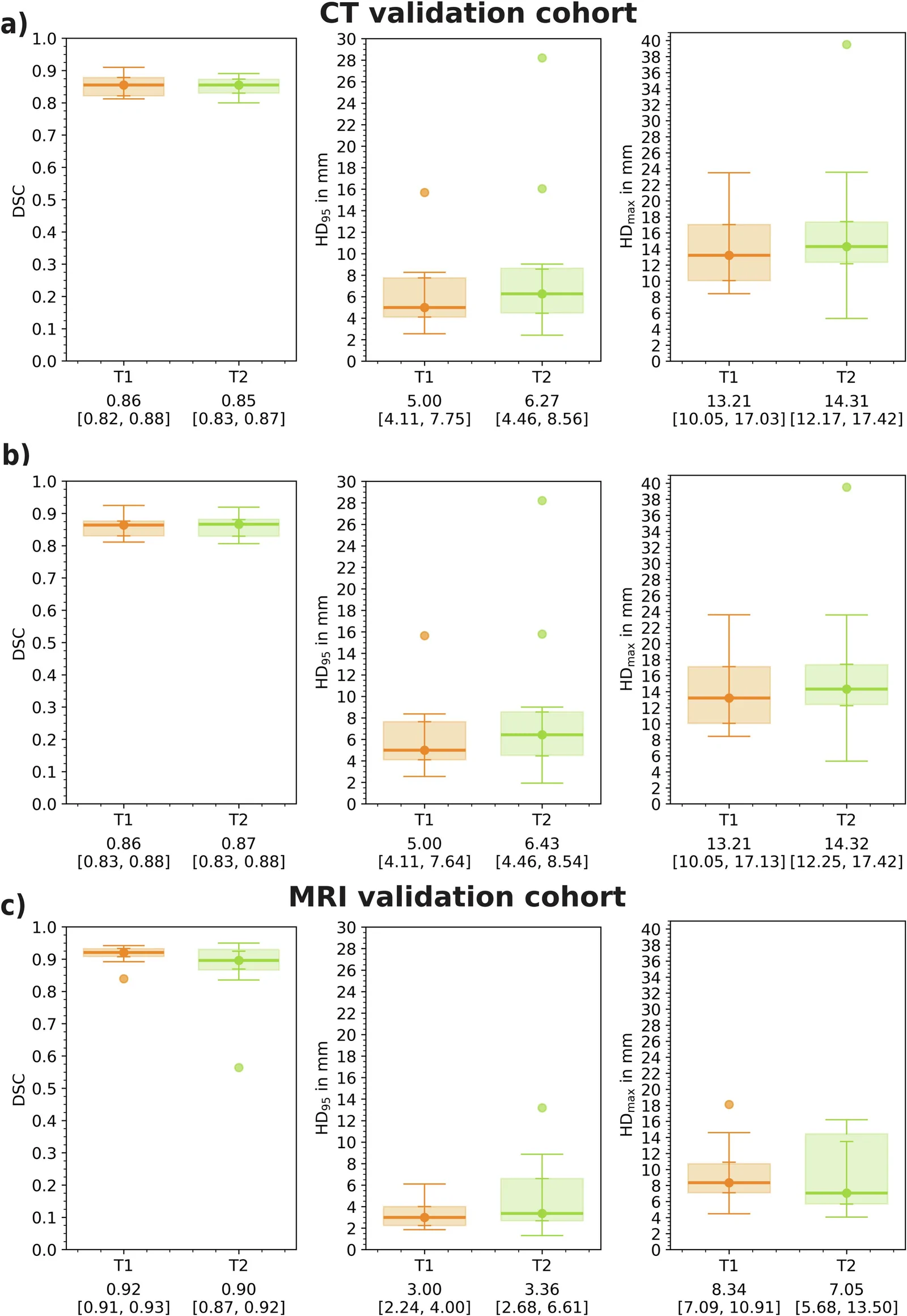
Boxplots of the brainstem segmentation measures–Dice similarity coefficient (DSC) and 100/95th-percentile Hausdorff distance (HD_max_/HD_95_)–for the T1 and T2 MRI weighting **a)** from the CT validation cohort after deformable registration with the brainstem as a focus region of interest and “mutual information” as the similarity measure; **b)** from the CT validation cohort after rigid registration; **c)** from the MR validation cohort. Boxes indicate the interquartile range ($Q_{1}-Q_{3}$), with the median shown as a horizontal line. Whiskers extend to 1.5 times the interquartile range below $Q_{1}$ and $Q_{3}$. Values beyond the whiskers are shown as outliers. Inner vertical bars indicate bootstrapped 95% confidence intervals of the median.

**Fig. 2:**
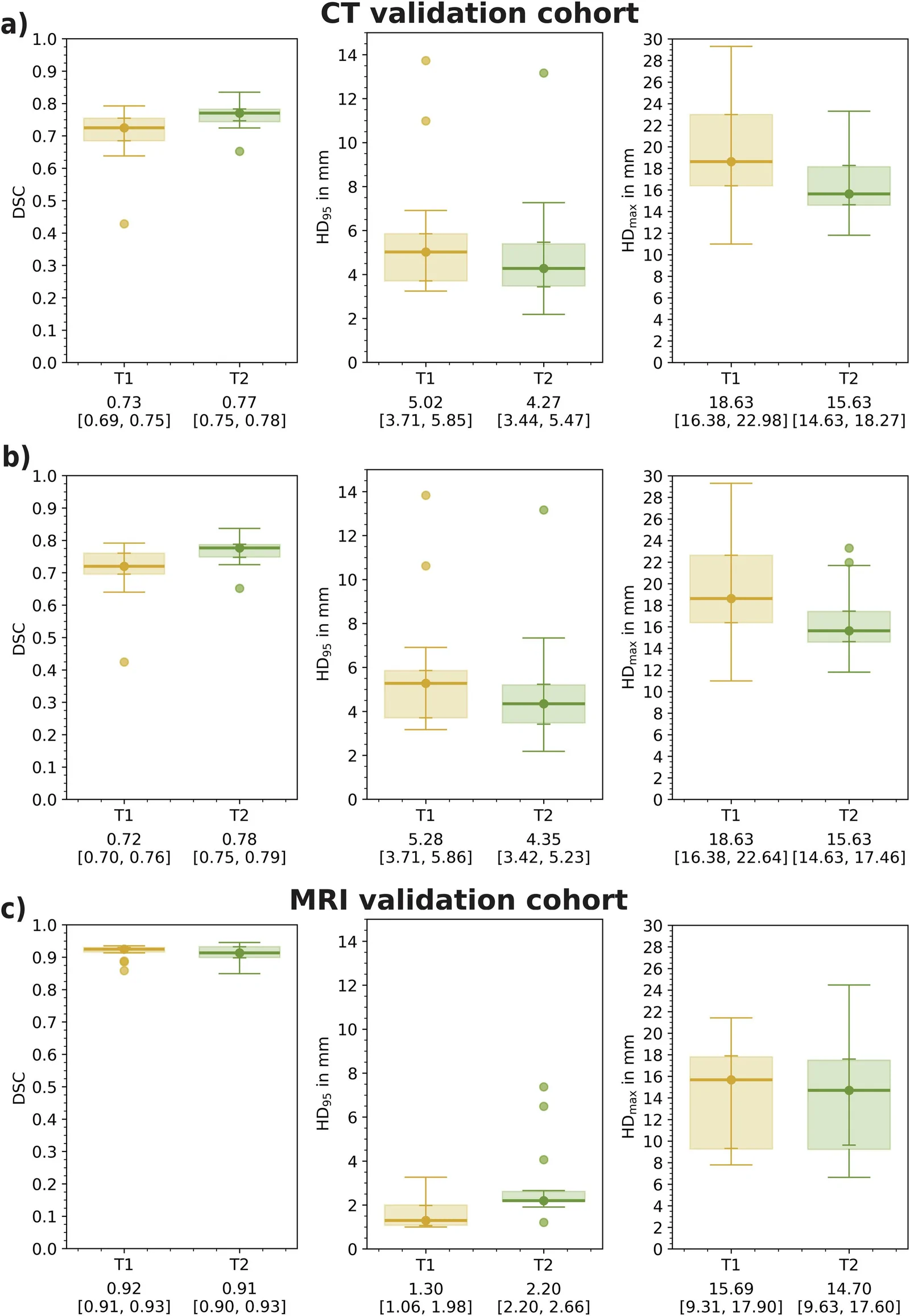
Boxplots of the ventricular system segmentation measures–Dice similarity coefficient (DSC) and 100/95th-percentile Hausdorff distance (HD_max_/HD_95_)–for the T1 and T2 MRI weighting **a)** from the CT validation cohort after deformable registration with the brainstem as a focus region of interest and “mutual information” as the similarity measure; **b)** from the CT validation cohort after rigid registration; **c)** from the MR validation cohort. Boxes indicate the interquartile range ($Q_{1}-Q_{3}$), with the median shown as a horizontal line. Whiskers extend to 1.5 times the interquartile range below $Q_{1}$ and $Q_{3}$. Values beyond the whiskers are shown as outliers. Inner vertical bars indicate bootstrapped 95% confidence intervals of the median.

No relevant grid-dependent differences from the MRI-grid results were observed (according to Table S3). Within the limitations of 14 cases per cohort, no statistically significant differences between the age groups were detected (see Figs. S1 and S2).

In routine clinical use, radiation oncologists estimated that the time required to obtain clinically acceptable VS and brainstem structures using the complete workflow, including DL-based segmentation, registration, contour mapping, and manual adjustments, was reduced by more than 50% compared with fully manual delineation. The DL-based segmentation step itself, i.e., inference with each model, required less than 1 min. For both prospective clinical cases (see Section 2.7), we rated the DL segmentation of the brainstem and VS with 4 to 5 on the 5-point Likert scale on the MRI and with 4 on the pCT (see further details in Table 2). Omission of the prepontine cistern in model-based VS segmentation was not an issue in the evaluation, as the training data likewise excluded this structure.

**Table 2:** 5-Point Likert scoring of the deep learning (DL)-generated ventricular system (target) and brainstem (organ of interest) segmentations across three criteria in prospective germ cell tumor cases. 1) Boundary Accuracy (BA): 1 = very poor (large mismatch/leakage); 2 = poor (major deviations); 3 = fair (notable deviations); 4 = good (minor fixes); 5 = excellent (near-perfect). 2) Anatomical Completeness (AC): 1 = very poor (major parts missing/mislabeled); 2 = poor (large gaps/spurious parts); 3 = fair (recognizable, some missing/spillover); 4 = good (minor gaps only); 5 = excellent (complete/correct). 3) Clinical Usability (CU): 1 = not usable; 2 = usable with extensive correction (>50%); 3 = moderate correction (∼20–50%); 4 = minor correction (<10–20%); 5 = fully usable (no correction). Note that the scoring of the ventricular system segmentation was performed without including the prepontine cistern.

		**5-point Likert score**				
**Structure**	**Criteria**	**1**	**2**	**3**	**4**	**5**
DL-generated brainstem	Boundary Accuracy			x		
	Anatomical Completeness				x	
	Clinical Usability			x		
DL-generated ventricular system	Boundary Accuracy				x	
	Anatomical Completeness				x	
	Clinical Usability				x	

In both cases, the brainstem—an important anatomical barrier for ventricles' delineation—was segmented in an anatomically plausible manner by the DL model (see Fig. S4). After mapping to the pCT, the pons was transferred essentially edit-free with a complete transfer. Limited, predictable adjustments on the pCT were needed (see Fig. 3), but resolved with minor cranial trimming, caudal elongation, and modest mediolateral smoothing. Deformable registration of the brainstem was not preferred in case 2 because it produced anatomically implausible warping in the medulla oblongata.

**Fig. 3:**
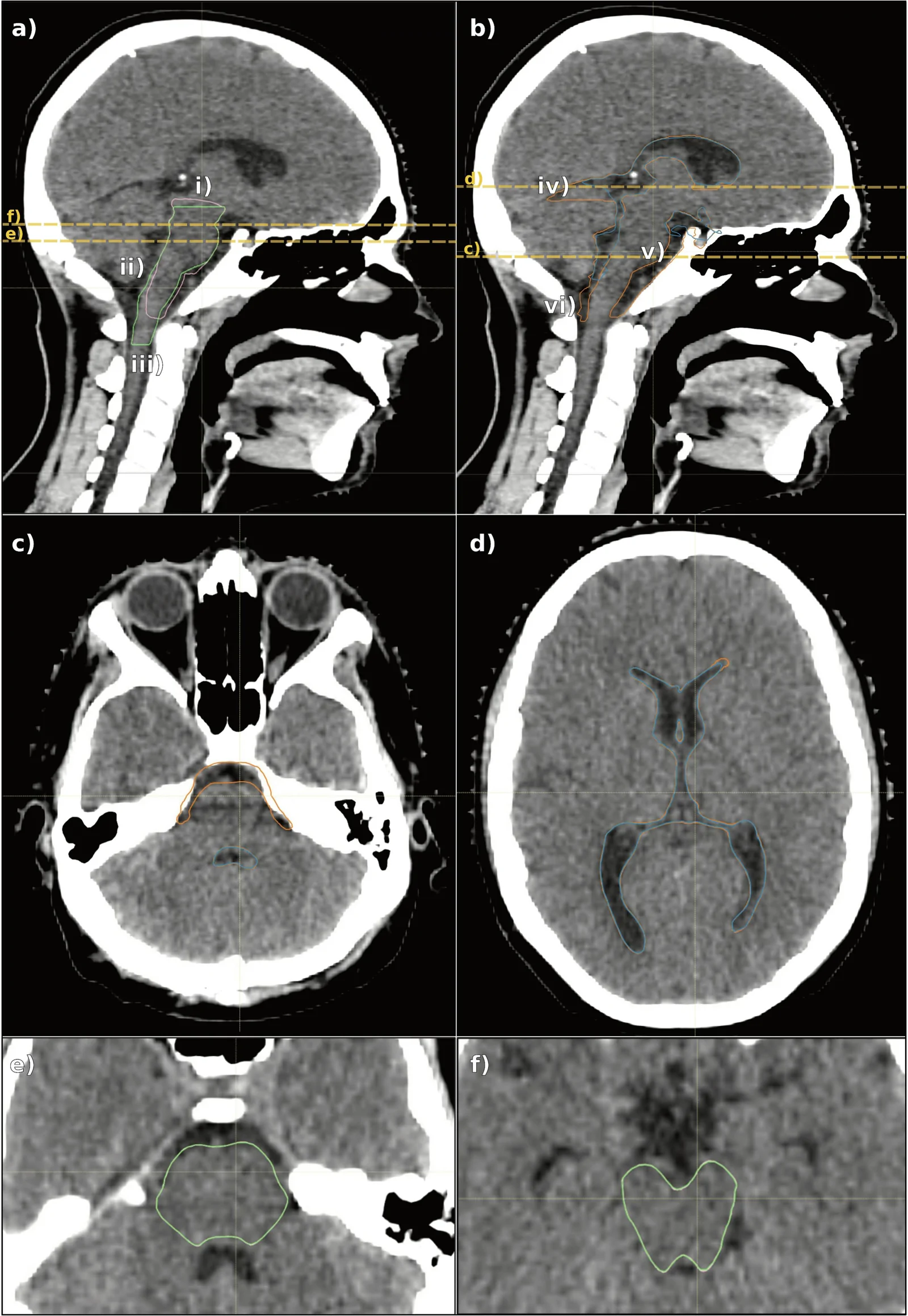
Comparison of the deep learning-generated structures mapped onto the planning CT (red: ventricular system; pink: brainstem) with the adapted and clinically approved structures (orange: ventricular system; green: brainstem). Sagittal view of the brain showing a) brainstem segmentation only and b) ventricular system segmentation only; transverse views showing c, d) ventricular systems only (at the prepontine cistern and lateral ventricle levels, respectively) and e, f) brainstem only (at the fourth ventricle and third ventricle levels, respectively). Adjustments have been applied in the following areas in addition to hole-filling in 3rd ventricle near pituitary stalk and a lateral smoothing at the pontocerebellar junction around the middle cerebellar peduncles: i) cranial brainstem trimming at cranial diencephalic-mesencephalic junction, ii) medulla oblongata shifting towards 4th ventricle, iii) inferior/sagittal extension of the medulla oblongata, iv) Cerebrospinal fluid contouring in the cerebellar region, v) manual contouring of prepontine cistern, vi) inferior/sagittal extension (≈5 mm) at 4th ventricle. (For interpretation of the references to colour in this figure legend, the reader is referred to the web version of this article.)

The DL-generated VS segmentation on MRI demonstrated high accuracy (see Fig. S3). For CT mapping, a brainstem-focused deformable registration was used. Mapped DL VS segmentations from different deformable-registration settings appeared broadly similar with minor shifts, while purely rigid registrations performed less accurately. In general, the mapped DL-generated VSs were particularly well contoured along the lateral ventricles, but also along the third ventricle and the floor of the third ventricle (see Fig. 3). Consistently, institution-specific refinements were nevertheless applied. The only notable exception was CSF extension at the cerebellar vermis in case 2, an anatomical peculiarity attributable to mass effect from the GCT located in the pineal region that exerted marked thalamic compression. This restraint in contouring was likewise reflected in the mapped VS structure.

## 4 Discussion

An MRI-based nnU-Net was implemented and evaluated in pediatric CNS tumor patients undergoing proton beam therapy. The DL contours were acceptable on MRI relative to manual references and clinically usable on pCT after deformable registration, with both findings supported by quantitative validation. These observations highlight the potential for immediate application in routine planning, with minimal need for manual adjustments. 

Accurate and consistent delineation of the VS and brainstem is critical in pediatric cranial RT, particularly given the risk of ventricular seeding and proximity of OoIs. Manual contouring is time-consuming and challenged by substantial ventricular inter- and intrapatient (time-dependent) shape variability [23], age-dependent anatomy, and clinician dependence. A robust automated approach could decrease planning time, streamline the planning workflow, and improve consistency. Given the close anatomical proximity and interdependence of the VS and brainstem, their consistent contouring enables them to accurately characterize their spatial relationship. This reduces inter-observer variability and enables a reliable management of target–OoI overlap [24], [25], [26], [27], which may eventually support sparing of the brainstem and PVR as OoIs. 

Quantitative head-to-head studies of manual versus DL-generated VS contours are scarce, reflecting the complexity of VS delineation and its recent indirect classification as a derived radiosensitive structure through its relationship to the PVR. Moreover, available studies typically do not train or evaluate segmentation models of the VS within an RT-specific workflow, or the underlying models are trained exclusively on adult cohorts. To the best of our knowledge, Wubbels et al. [28] were the first to clinically evaluate (CT-space) DL-based segmentation of the entire VS against manual contours, using the 2021 EPTN atlas [1] and likewise excluding the prepontine cistern. However, the study was restricted to adult patients. A superior segmentation performance of nnU-Net compared with SynthSeg segmentations integrated into FreeSurfer (v7.3.2) was reported [28]. The segmentation performance, with a DSC of 0.93 [0.86–0.95] and an HD_95_ of 0.9 [0.7–2.5] mm, was comparable to the performance observed in our MR cohort, which achieved DSC values of 0.92 [0.91–0.93] for T1wIs and 0.91 [0.90–0.93] for T2wIs, and HD_95_ values of 1.3 [1.1–2.1] mm for T1wIs and 2.2 [2.2–2.7] mm for T2wIs [28]. Their local surface-variation analysis also revealed increased variability in the temporal and occipital horns of the lateral ventricles, whereas the most pronounced deviations in our cohort were located near the outlets of the fourth ventricle. By contrast, available brainstem benchmarks provide context for the results of this study, with adult studies reporting median DSC scores ≤0.9 and others reporting interobserver variability in a similar range to the segmentation performance observed in both our cohorts (see Table 3). However, HD-based brainstem metrics were larger in the CT-space end-to-end workflow cohort than in the MR cohort. Against this backdrop, the models' results fall within clinically acceptable ranges, supporting deployment in treatment planning, provided that routine clinical review is retained. Nevertheless, isolated outliers in HDs indicate that clinically relevant local deviations occurred in individual cases.

**Table 3:** Adult studies about brainstem contouring: comparing deep learning-generated brainstem contours with the reference manual brainstem contours or evaluating interobserver variation of brainstem contouring on CT images. For comparison with our study with pediatric patients (see first four rows including the T1-weighted MRI (T1wI)- and T2-weighted MRI (T2wI)-based segmentation results), only the measures Dice similarity coefficient (DSC), 100 and 95th-percentile Hausdorff distance (HD_95%,_ and HD_max_) are listed with the median value and the range with min and max values in parentheses or the interquartile range in brackets if not otherwise specified. The study [28] for adults about the deep learning-generated ventricular system did not list any evaluation measures.

**Evaluation focus**	**Reference**	**Manual contouring**	**Imaging modality of comparison**	**DSC**	**HD**_**95%**_**(mm)**	**HD**_**max**_**(mm)**
Comparison of DL and manual contours	this work	One physician and one physicist, and one radiation oncologist for approval	T1wI	0.92 [0.91–0.93] Mean ± std.: 0.92 ± 0.03	3.00 [2.22–4.00] Mean ± std.: 3.34 ± 1.39	8.34 [7.09–10.91] Mean ± std.: 9.35 ± 3.76
			T2wI	0.90 [0.86–0.94] Mean ± std.: 0.88 ± 0.10	3.36 [2.68–6.61] Mean ± std.: 4.88 ± 3.46	7.05 [5.68–15.49] Mean ± std.: 9.36 ± 4.73
		Several radiation oncologists and observers for final approval contoured for clinical cases over time	CT (with T1wI-based DL contours)	0.86 [0.82–0.88] Mean ± std.: 0.85 ± 0.03	5.00 [4.00–7.84] Mean ± std.: 6.13 ± 3.47	13.21 [10.01–17.14] Mean ± std.: 13.98 ± 4.51
			CT (with T2wI-based DL contours)	0.85 [0.83–0.87] Mean ± std.: 0.85 ± 0.03	6.27 [4.46–8.83] Mean ± std.: 8.16 ± 6.67	14.31 [12.17–17.42] Mean ± std.: 16.09 ± 7.99
Studies comparing DL and manual contours	Turcas et al. [29] (auto-segmentation with software MVision GBS™, Version 1.2.2, CNN-based)	One radiation oncologist (contoured study-specific)	CT (with T1wI-based DL contours)	0.90 [0.90–0.94]	/	6.97 [5.03–8.70]
	Gibbons et al. [30] (auto-segmentation with software, DLCExpert, CNN-based)	One radiation oncologist (contoured study-specific)	CT	0.85 [0.83–0.87]	/	10.04 [8.9–11.6]
	Chen et al. [31] (auto-segmentation with RayStation deep learning tool v2.0.0.45, 3D CNN U-Net-based)	Several radiation oncologists and radiation technologists contoured for clinical cases over time (retrospective)	CT	Mean ± std.: 0.9 ± 0.1	Mean ± std.: 4.2 ± 1.9	/
Studies evaluating interobserver variation	Nelms et al. [32]		CT	Mean ± std.: 0.66 ± 0.17	/	/
	Nielsen et al. [33]		CT	0.82 [0.78–0.84]	4.6 [4.0–5.6]	8.1 [7.1–9.6]
	Wong et al. [34]		CT	Worst mean 0.78	Worst mean 4.01	/
	Van der Veen et al. [35]		CT	0.88 (0.61–0.92)	4.0 (2.3–15.0)	/

Lower DSC values and higher boundary distances in the CT validation cohort were expected and are attributable to several factors. First, routine clinical pCT contours do not constitute a strict ground truth: the VS was drawn conservatively and generously because of time constraints and because clinical target definitions typically included a protocol-specific margin around the VS. Second, the dataset spanned from 2017 to 2025 and involved multiple radiation oncologists with varying levels of experience, introducing interobserver variability [27], [36]. These may be more pronounced for fine-border structures such as the VS and are still relevant for the brainstem, which is consistent with reported ranges (Table 3). Third, the evaluation on CTs required registration-based mapping from MRI. Residual rigid or deformable misalignment inflates Hausdorff distances and can depress DSC, particularly at interfaces sensitive to posture or sequence contrast. Fourth, manual contours themselves are constrained by MRI and CT imaging quality—acquisition parameters, contrast, resolution, and artifacts—so comparisons between manual and DL segmentations measure agreement under the same image limitations rather than truth in an absolute sense. Finally, definitions of radiotherapy OoIs, such as the brainstem, vary across protocols, adding heterogeneity.

Conversely, higher DSC values and lower Hausdorff metrics in the MRI validation cohort are readily explained by methodological and imaging factors. Manual reference labels were generated by the same two raters and approved by a third rater, thereby mitigating interobserver variability, though intraobserver variation remains possible [36]. MRI comparisons did not require mapping to CT, thereby avoiding uncertainties associated with registration.

Registration accuracy and the fidelity of mapped contours depend strongly on acquisition quality [37]. Important determinants are signal-to-noise ratio, CSF–parenchyma contrast-to-noise ratio, spatial resolution, and geometric fidelity. Higher in-plane resolution and thin, near-cubic voxels reduce partial-volume effects at thin ependymal surfaces and high-curvature boundaries (e.g., fastigium, aqueduct), stabilizing both the learned boundary and the registration cost function [38], [39]. Conversely, anisotropic sampling with few, thick slices produces staircase artifacts after resampling, degrading surface distances (increasing HD_95_/HD_max_) even when the DSC remains moderate. In the CT-validation cohort, T2wIs and T1wIs had median z-direction slice counts of 79 and 200 and slice spacings of 2.2 mm and 1.0 mm, respectively, with a 2.0-mm slice thickness for both. In addition to differences in slice sampling, variations in MRI quality and sequence-dependent contrast affect MRI-based segmentation, downstream registration, and contour mapping.

Without mapping, the DL model performed particularly well along the lateral ventricles and the floor of the third ventricle and produced stable brainstem contours in regions with clear CSF interfaces. After mapping to the pCT, the agreement remained high in most regions. A lower performance was observed where registration is inherently more difficult, such as across posterior-fossa transitions along the medulla oblongata and near the cerebellar vermis and fourth-ventricle outlets. Here, immobilization-related, large posture differences, e.g., thermoplastic-mask–induced neck inclination at pCT versus pMRI acquired without immobilization aids, specifically alter brainstem geometry and surrounding tissue. However, within the skull, anatomical deformations remain small, allowing for accurate rigid registration between CT and MR images [37], [40]. Where available, MR simulation could replicate pCT treatment positioning and immobilization, thereby reducing registration uncertainties [41], [42], [43]. Furthermore, images acquired on the in-house Toshiba Vantage Titan Large Bore 1.5 T scanner did not undergo additional geometric MRI distortion correction. These distortions—albeit small for pediatric head scans—may constitute a non-negligible source of spatial misalignment and may not be compensated by subsequent deformable registration. Due to these dependencies, two practical strategies appear reasonable for mapping DL contours from MRI to CT for integration into the current clinical workflow: mapping of VS and brainstem using a single, well-vetted registration when posture is comparable, or mapping with structure-specific registrations when posture or local deformations differ. But in all cases, accurate frame-of-reference rigid alignment is an indispensable prerequisite before applying non-rigid refinement.

The limited cohort size constrained both training and validation. Larger datasets across scanners, institutions, and patient populations would have increased diversity and improved generalizability, but their collection is challenging due to the labor-intensive manual contouring and post-segmentation quality review required. However, both the training and test cohorts encompassed substantial anatomical variability and covered the full pediatric age range. As GCTs typically occur beyond early childhood, the GCT-CT cohort mainly included older children but still covered a broad pediatric age range. The models therefore appear robust within pediatric populations, whereas adult use remains investigational until adult cases are included in training and validation.

Model optimization and performance evaluation during training relied on the DSC, which may underemphasize boundary accuracy. Future studies should investigate interobserver variability for both structures on MRI and, in particular, for VS on CT.

In conclusion, we introduced MRI-based DL models for VS and brainstem segmentation in pediatric PBT. The models demonstrated high performance on MRI and, after registration, on CT across patients with various tumor histologies and substantial variability in the anatomy of the VS and brainstem. Although required contour adjustments on pCT for clinical acceptance were minimal and easily manageable within RS, consistent with findings from other segmentation studies [44], routine visual inspection and, where necessary, manual correction remain essential before clinical use. While the prepontine cistern has been formally incorporated into the VS definition in the current atlas since 2025 [45], it is straightforward to localize and can be delineated with minimal additional effort. Automatic WVV segmentation reduced contouring workflow time by ≈50%, streamlining the planning process.

## Declaration of generative AI and AI-assisted technologies in the manuscript preparation process

During the preparation of this work, the authors used GPT-5.2 in order to improve the readability and language of the manuscript. After using this service, the authors reviewed and edited the content as needed and take full responsibility for the content of the published article.

## CRediT authorship contribution statement

**Feline Heinzelmann:** Writing – review & editing, Writing – original draft, Visualization, Validation, Software, Methodology, Investigation, Formal analysis, Data curation, Conceptualization. **Fabian Hörst:** Software, Methodology. **Lukas Heine:** Software, Methodology. **Jana Fragemann:** Writing – review & editing. **Moritz Rempe:** Software, Methodology. **Gijs Luijten:** Methodology. **Sarah Peters:** Writing – review & editing, Validation, Conceptualization. **Julian Hlouschek:** Writing – review & editing, Validation. **Jan Egger:** Supervision. **Beate Timmermann:** Writing – review & editing, Resources, Funding acquisition. **Jens Kleesiek:** Writing – review & editing, Supervision, Resources, Funding acquisition. **Christian Bäumer:** Writing – review & editing, Supervision, Resources, Methodology, Funding acquisition, Conceptualization.

## Declaration of competing interest

The authors declare that they have no known competing financial interests or personal relationships that could have appeared to influence the work reported in this paper.
