# Supplementary material for: Deep learning segmentation of the cerebral ventricular system and brainstem for pediatric radiotherapy planning
Source: Phys Imaging Radiat Oncol. 2026 Sep 19;41:101086. doi: 10.1016/j.phro.2026.101086 (PMC13651686; doi:10.1016/j.phro.2026.101086)
Supplement: Supplementary file 1 [file mmc1.pdf]

Supplementary material

Table S1. Mean Dice similarity coefficient values computed from each cross-validation prediction for nnU-Net-based segmentation of the ventricular system and brainstem and averaged Dice similarity coefficients across all folds for each trained model and corresponding standard deviations (Std). The "2D-nnU-Net" models with T1- and T2-weighted images as input were selected as the final deep learning segmentation models for inference.

Input: T1	3D-nnU-Net							2D-nnU-Net						
Fold	0	1	2	3	4	Mean	Std	0	1	2	3	4	Mean	Std
Ventricular system	0.912	0.880	0.850	0.791	0.836	0.854	0.046	0.918	0.854	0.857	0.788	0.879	0.859	0.047
Brainstem	0.930	0.939	0.932	0.929	0.936	0.933	0.004	0.926	0.940	0.930	0.932	0.942	0.934	0.007
Mean	0.921	0.910	0.891	0.860	0.886			0.922	0.897	0.893	0.860	0.910		

Input: T2	3D-nnU-Net							2D-nnU-Net						
Fold	0	1	2	3	4	Mean	Std	0	1	2	3	4	Mean	Std
Ventricular system	0.905	0.873	0.857	0.771	0.886	0.858	0.052	0.897	0.869	0.857	0.767	0.873	0.853	0.050
Brainstem	0.920	0.922	0.927	0.924	0.920	0.922	0.003	0.914	0.921	0.921	0.916	0.921	0.918	0.004
Mean	0.913	0.898	0.892	0.847	0.903			0.905	0.895	0.889	0.841	0.897		

Input: T1T2	3D-nnU-Net							2D-nnU-Net						
Fold	0	1	2	3	4	Mean	Std	0	1	2	3	4	Mean	Std
Ventricular system	0.911	0.903	0.854	0.841	0.834	0.869	0.036	0.885	0.843	0.854	0.840	0.832	0.851	0.021
Brainstem	0.930	0.935	0.930	0.928	0.936	0.932	0.003	0.923	0.939	0.929	0.923	0.935	0.930	0.007
Mean	0.921	0.919	0.892	0.885	0.885			0.904	0.891	0.892	0.882	0.884		

Input: T2T1	3D-nnU-Net							2D-nnU-Net						
Fold	0	1	2	3	4	Mean	Std	0	1	2	3	4	Mean	Std
Ventricular system	0.907	0.898	0.851	0.773	0.858	0.857	0.053	0.897	0.900	0.851	0.761	0.860	0.854	0.056
Brainstem	0.921	0.922	0.935	0.926	0.918	0.925	0.007	0.907	0.927	0.924	0.908	0.913	0.916	0.009
Mean	0.914	0.910	0.893	0.850	0.888			0.902	0.913	0.888	0.835	0.886		

Abbreviations: VS, ventricular system; std, standard deviation

Table S2. NIfTI conversion statistics for the T1- and T2-weighted MR images used for training within the nnU-Net framework.

**T1-weighted MR images**			**T2-weighted MRI images**		
	**Median spacing (mm)**	1.000<br>0.449<br>0.449		**Median spacing (mm)**	2.200<br>0.413<br>0.413
	**Median shape**	192<br>525<br>483		**Median shape**	63<br>555<br>499
**2D configuration**	**Batch size**	12	**2D configuration**	**Batch size**	12
	**Patch size**	512<br>512		**Patch size**	512<br>512
	**Median image size in voxels**	492.0<br>456.0		**Median image size in voxels**	532.0<br>486.0
	**Spacing (mm)**	0.449<br>0.449		**Spacing (mm)**	0.413<br>0.413
**3D low resolution**	**Batch size**	2	**3D low resolution**	**Batch size**	2
	**Patch size**	80<br>192<br>160		**Patch size**	40<br>224<br>224
	**Median image size in voxels**	127<br>289<br>268		**Median image size in voxels**	63<br>373<br>341
	**Spacing (mm)**	1.513<br>0.764<br>0.764		**Spacing (mm)**	2.200<br>0.589<br>0.589
**3D full resolution**	**Batch size**	2	**3D full resolution**	**Batch size**	2
	**Patch size**	64<br>192<br>160		**Patch size**	32<br>256<br>224
	**Median image size in voxels**	192.0<br>492.0<br>456.0		**Median image size in voxels**	63.0<br>532.0<br>486.0
	**Spacing (mm)**	1.000<br>0.449<br>0.449		**Spacing (mm)**	2.200<br>0.413<br>0.413

Table S3. Difference and equivalence scenarios for comparisons between fixed target grid resolutions and the patient-specific MRI grid used as reference.
p_{Holm} represents the p-value from the paired Wilcoxon signed-rank test after Holm correction for multiple comparisons within each metric ($m = 5$, corresponding to the target grid resolutions 0.25 mm, 0.5 mm, 0.75 mm, 1 mm, and 2 mm). p_{TOST} represents the maximum p-value from the paired TOST equivalence test. p_{Holm} tests whether the target grid differs significantly from the MRI grid, whereas p_{TOST} tests whether the difference is statistically contained within the predefined equivalence margin. Scenario 3 represents the least favorable outcome when assessing grid equivalence, indicating that the target grid differed significantly from the MRI grid and that equivalence within the predefined margin could not be demonstrated. The least favorable scenario (Scenario 3) occurred only when comparing the 0.25-mm target grid with the patient-specific MRI grid, namely for HD95 in the T1 ventricle analysis and for DSC in the T2 ventricle analysis.

**Scenario**	p_{Holm}	p_{TOST}	**Interpretation**
**1**	< 0.05	< 0.05	The difference is statistically significant but lies within the equivalence margin.
**2**	≥ 0.05	< 0.05	No statistically significant difference was detected, and equivalence is demonstrated.
**3**	< 0.05	≥ 0.05	A statistically significant difference was detected, and equivalence is not demonstrated.
**4**	≥ 0.05	≥ 0.05	No statistically significant difference was detected, but equivalence is also not demonstrated; the result is inconclusive with respect to equivalence.

Table S4. General 5-point Likert score to evaluate the prospective clinical cases qualitatively.

Score	Meaning	Detailed meaning
**1**	**Very Poor**	• Segmentation grossly inaccurate.<br>• Large parts of the structure missing or mislabeled.<br>• Severe leakage into non-target tissue.<br>• Not usable for any analysis or treatment planning.
**2**	**Poor**	• Overall shape partly recognizable, but with major errors (e.g., truncated boundaries,<br>• over-segmentation).<br>• Requires extensive manual correction (>50% of structure).<br>• Not acceptable for clinical use.
**3**	**Fair**	• Anatomical structure reasonably outlined but with clear inaccuracies.<br>• Boundaries not reliable, some missing regions or spillover.<br>• Usable only with substantial corrections.
**4**	**Good**	• Structure well represented, correct overall shape and location.<br>• Only minor boundary adjustments needed (<10–20%).<br>• Acceptable for most research or clinical workflows.
**5**	**Excellent**	• Segmentation anatomically accurate and complete.<br>• Boundaries align with expert expectation.<br>• No or negligible corrections required.<br>• Fully usable for clinical/research purposes.

Table S5. Detailed 5-point Likert Score to evaluate the prospective clinical cases qualitatively.

**Boundary Accuracy**	**How well do the segmentation boundaries align with the true anatomical borders?**
**Score**	**1** = Very poor, large mismatches, severe leakage or truncation<br>**2** = Poor, major boundary errors<br>**3** = Fair, boundaries roughly follow anatomy but with notable deviations<br>**4** = Good, minor boundary corrections needed<br>**5** = Excellent, boundaries match anatomy almost perfectly
**Anatomical Completeness**	**Is the structure fully represented, without missing or spurious parts?**
**Score**	**1** = Very poor, major parts missing or mislabeled<br>**2** = Poor, large regions incomplete or with spurious extensions<br>**3** = Fair, structure recognizable but with missing regions or spillover<br>**4** = Good, mostly complete with only minor corrections needed<br>**5** = Excellent, complete and anatomically correct
**Clinical Usability**	**How usable is this segmentation for clinical/research applications without manual correction?**
**Score**	**1** = Not usable at all<br>**2** = Usable only with extensive correction (>50%)<br>**3** = Usable with moderate correction (~20–50%)<br>**4** = Usable with minor corrections (<10–20%)<br>**5** = Fully usable, no corrections needed

Table S6. Segmentation measures from the CT validation cohort comparing the deep learning-generated structures (brainstem and ventricular system) from the T1-/T2-weighted MRI with the manual segmented structures on the planning CT. The structures are evaluated on a voxelized 3D grid with the pixel spacing from the patient's planning CT.

	Reg	Metric	Weighting	*n*	Mean	Std	Median	25 th perc	75 th perc	IQR	Min	Max	95% CI
Brainstem	deformable	DSC	T1	13	0.853	0.033	0.855	0.821	0.880	0.059	0.812	0.910	[0.822, 0.878]
			T2	14	0.851	0.029	0.855	0.830	0.874	0.044	0.800	0.891	[0.830, 0.873]
		HD_{95} (mm)	T1	13	6.127	3.468	5.000	4.002	7.844	3.842	2.548	15.691	[4.110, 7.754]
			T2	14	8.160	6.671	6.274	4.460	8.835	4.375	2.423	28.219	[4.460, 8.559]
		HD_{max} (mm)	T1	13	13.980	4.507	13.205	10.006	17.144	7.138	8.418	23.509	[10.048, 17.033]
			T2	14	16.086	7.991	14.312	12.168	17.423	5.255	5.339	39.506	[12.168, 17.423]
	rigid	DSC	T1	13	0.859	0.035	0.864	0.828	0.880	0.053	0.811	0.925	[0.830, 0.877]
			T2	14	0.858	0.033	0.867	0.829	0.883	0.053	0.806	0.919	[0.829, 0.881]
		HD_{95} (mm)	T1	13	6.116	3.456	5.000	4.049	7.763	3.714	2.548	15.654	[4.110, 7.636]
			T2	14	8.134	6.667	6.427	4.460	8.723	4.263	1.933	28.220	[4.460, 8.543]
		HD_{max} (mm)	T1	13	13.982	4.500	13.205	10.006	17.216	7.210	8.418	23.596	[10.048, 17.128]
			T2	14	16.082	7.984	14.321	12.248	17.423	5.175	5.339	39.506	[12.248, 17.423]
Ventricular system	deformable	DSC	T1	13	0.700	0.094	0.725	0.675	0.757	0.082	0.428	0.793	[0.685, 0.755]
			T2	14	0.766	0.046	0.770	0.741	0.783	0.043	0.653	0.835	[0.747, 0.783]
		HD_{95} (mm)	T1	13	5.845	3.156	5.021	3.670	6.114	2.444	3.242	13.729	[3.706, 5.849]
			T2	14	4.997	2.716	4.270	3.418	5.467	2.049	2.181	13.166	[3.437, 5.467]
		HD_{max} (mm)	T1	13	19.381	4.933	18.627	16.041	23.111	7.070	10.992	29.306	[16.385, 22.983]
			T2	14	16.679	3.451	15.631	14.308	18.275	3.967	11.807	23.294	[14.628, 18.275]
	rigid	DSC	T1	13	0.702	0.095	0.720	0.686	0.761	0.076	0.424	0.791	[0.696, 0.761]
			T2	14	0.769	0.046	0.777	0.748	0.788	0.040	0.652	0.837	[0.748, 0.788]
		HD95 (mm)	T1	13	5.866	3.119	5.277	3.670	6.122	2.453	3.169	13.829	[3.706, 5.859]
			T2	14	4.966	2.720	4.349	3.418	5.229	1.811	2.181	13.166	[3.418, 5.229]
		HDmax (mm)	T1	13	19.287	4.926	18.627	16.041	22.727	6.686	10.992	29.306	[16.385, 22.641]
			T2	14	16.648	3.508	15.631	14.308	17.462	3.155	11.807	23.294	[14.628, 17.462]

Table S7. Segmentation measures from the MRI validation cohort comparing the deep learning-generated structures with the manual segmented structures (brainstem and ventricular system) on the T1-/T2-weighted MRI. The structures are evaluated on a voxelized 3D grid with the pixel spacing from the patient's respective MRI.

ROI	Metric	Weighting	*n*	Mean	Std	Median	25 th perc	75 th perc	IQR	Min	Max	95% CI
**Brainstem**	**DSC**	T1	14	0.916	0.026	0.921	0.907	0.933	0.026	0.839	0.942	[0.908, 0.933]
		T2	14	0.876	0.096	0.896	0.862	0.936	0.073	0.564	0.950	[0.869, 0.925]
	**HD_{95} (mm)**	T1	14	3.339	1.389	3.000	2.217	4.000	1.783	1.855	6.098	[2.238, 4.000]
		T2	14	4.882	3.461	3.364	2.678	6.611	3.933	1.307	13.200	[2.678, 6.611]
	**HD_{max} (mm)**	T1	14	9.352	3.759	8.341	7.090	10.911	3.821	4.474	18.117	[7.090, 10.911]
		T2	14	9.359	4.732	7.055	5.677	15.485	9.808	4.052	16.199	[5.677, 13.496]
**Ventricular system**	**DSC**	T1	14	0.917	0.023	0.925	0.913	0.930	0.017	0.859	0.935	[0.913, 0.930]
		T2	14	0.909	0.030	0.913	0.898	0.933	0.036	0.849	0.945	[0.898, 0.932]
	**HD_{95} (mm)**	T1	14	1.621	0.699	1.298	1.064	2.052	0.989	1.000	3.265	[1.064, 1.979]
		T2	14	2.952	1.802	2.200	2.200	2.657	0.457	1.208	7.378	[2.200, 2.657]
	**HD_{max} (mm)**	T1	14	14.355	4.880	15.686	8.923	18.061	9.138	7.791	21.424	[9.315, 17.896]
		T2	14	14.086	5.333	14.700	8.800	17.600	8.800	6.644	24.467	[9.626, 17.600]

Table S8. Volume for each structure and cohort and volume difference Δ between manual and deep-learning generated structure. Note that the manual structures are the same for the CT cohort when comparing with the DL structures mapped from T1-weighted MRIs or from T2-weighted MRIs. Since there are 14 cases included for DL structures mapped from T2-weighted MRIs and 13 cases mapped from T1-weighed MRIs, the values differ in the volumes of "Manual BS" and "Manual VS" from the CT validation cohort.

Validation cohort	MRI weighting	BS volume (cm^3)	Mean	Median	Min	Max	*n*
CT	T1	**Manual BS**	23.183	23.670	17.923	28.377	13
		**DL BS**	22.607	22.448	16.335	29.016	13
		Δ (%)	-2.073	-0.992	-19.925	15.318	13
	T2	**Manual BS**	22.991	22.819	17.923	28.377	14
		**DL BS**	21.721	22.160	16.776	28.381	14
		Δ	-5.028	-6.002	-20.794	11.576	14
MRI	T1	**Manual BS**	24.038	23.553	18.777	32.005	14
		**DL BS**	21.762	22.327	16.342	25.779	14
		Δ	-9.045	-10.787	-26.313	15.529	14
	T2	**Manual BS**	23.763	23.688	18.327	33.062	14
		**DL BS**	19.915	20.294	13.259	24.701	14
		Δ	-14.604	-12.805	-59.895	11.022	14
**Validation cohort**	**MRI weighting**	**VS volume (cm^3)**	**Mean**	**Median**	**Min**	**Max**	***n***
CT	T1	**Manual VS**	48.352	48.198	34.956	60.568	13
		**DL VS**	32.808	29.861	18.447	52.883	13
		Δ	-32.648	-31.612	-47.226	-2.775	13
	T2	**Manual VS**	48.194	47.723	34.956	60.568	14
		**DL VS**	37.099	32.954	19.319	61.699	14
		Δ	-23.927	-27.418	-44.732	13.434	14
MRI	T1	**Manual VS**	59.138	61.189	30.853	99.652	14
		**DL VS**	56.155	60.017	28.182	94.103	14
		Δ	-5.220	-5.088	-14.821	1.542	14
	T2	**Manual VS**	69.387	71.040	39.339	102.707	14
		**DL VS**	66.753	69.607	35.976	107.198	14
		Δ	-3.807	-3.453	-18.431	4.373	14

Abbreviations: CI, confidence interval; n, number; CT, computed tomography; MRI, magnet resonance imaging; VS, ventricular system; BS, brainstem.

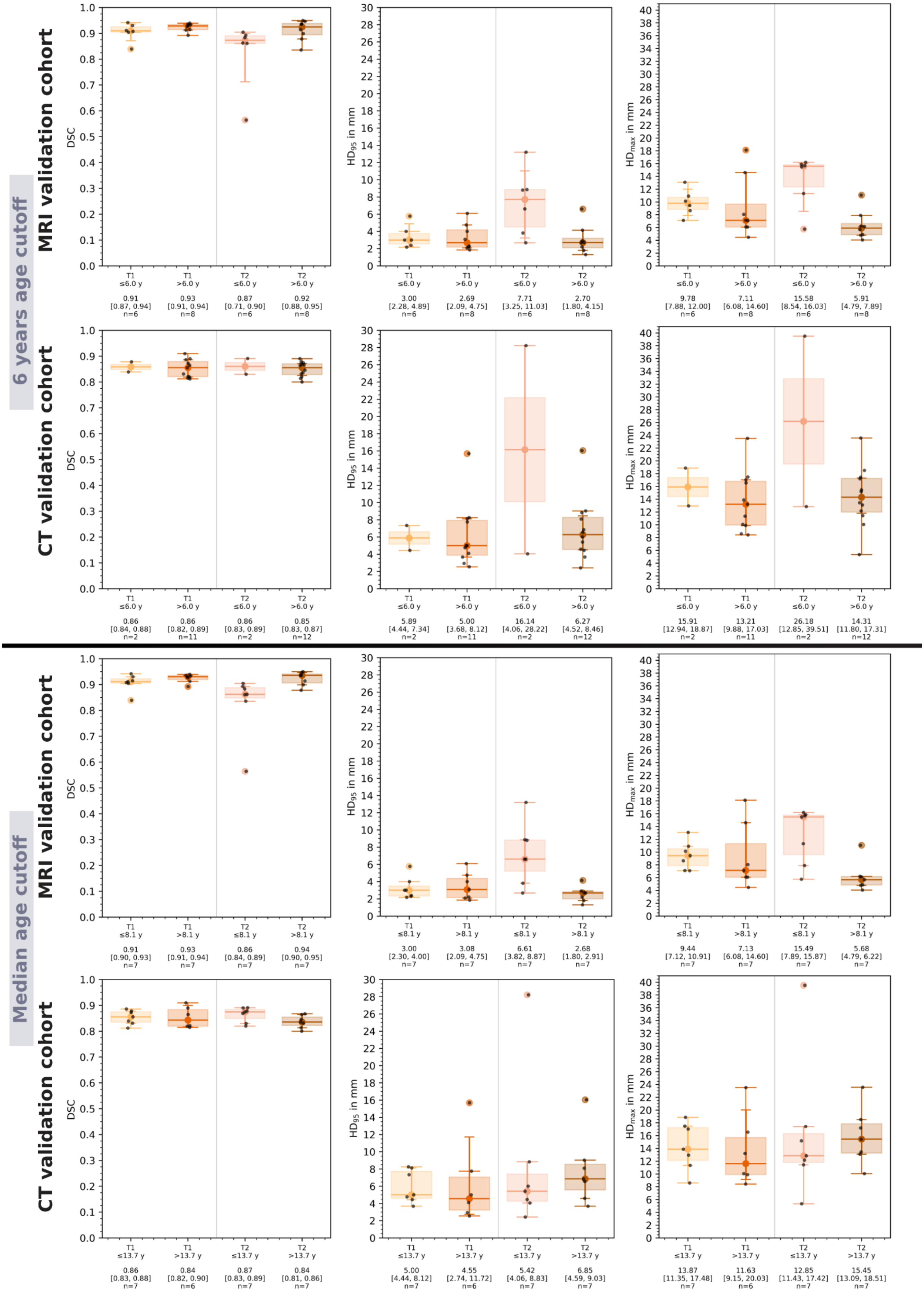

Figure S1. Age comparison with boxplots of the brainstem segmentation measures–Dice similarity coefficient (DSC) and 100th/95th-percentile Hausdorff distance (HD_{max}/HD_{95})–for the T1 and T2 MRI weighting for the MR validation cohort and for the CT validation cohort after deformable registration with the brainstem as a focus region of interest and "mutual information" as the similarity measure. Above the thick black horizontal line: 6 years as age cutoff; below the thick black horizontal line: median age (of the respective validation cohort) as age cutoff. Boxes represent the interquartile range (IQR,Q_1-Q_2) with the median as a horizontal line. Whiskers extend to $[L, U] = [Q_1 - 1.5 \times \text{IQR}, Q_3 + 1.5 \times \text{IQR}]$; more extreme values are plotted as outliers $O = \{x_i : x_i < L \text{ or } x_i > U\}$. Inner vertical bars indicate bootstrapped 95% confidence intervals (CI) of the median. Due to the small number of cases per age group, the raw data for each case are shown as black scatter points. Where differences were statistically significant according to the Mann–Whitney U test, significance was indicated in the boxplot as $p < 0.05$.

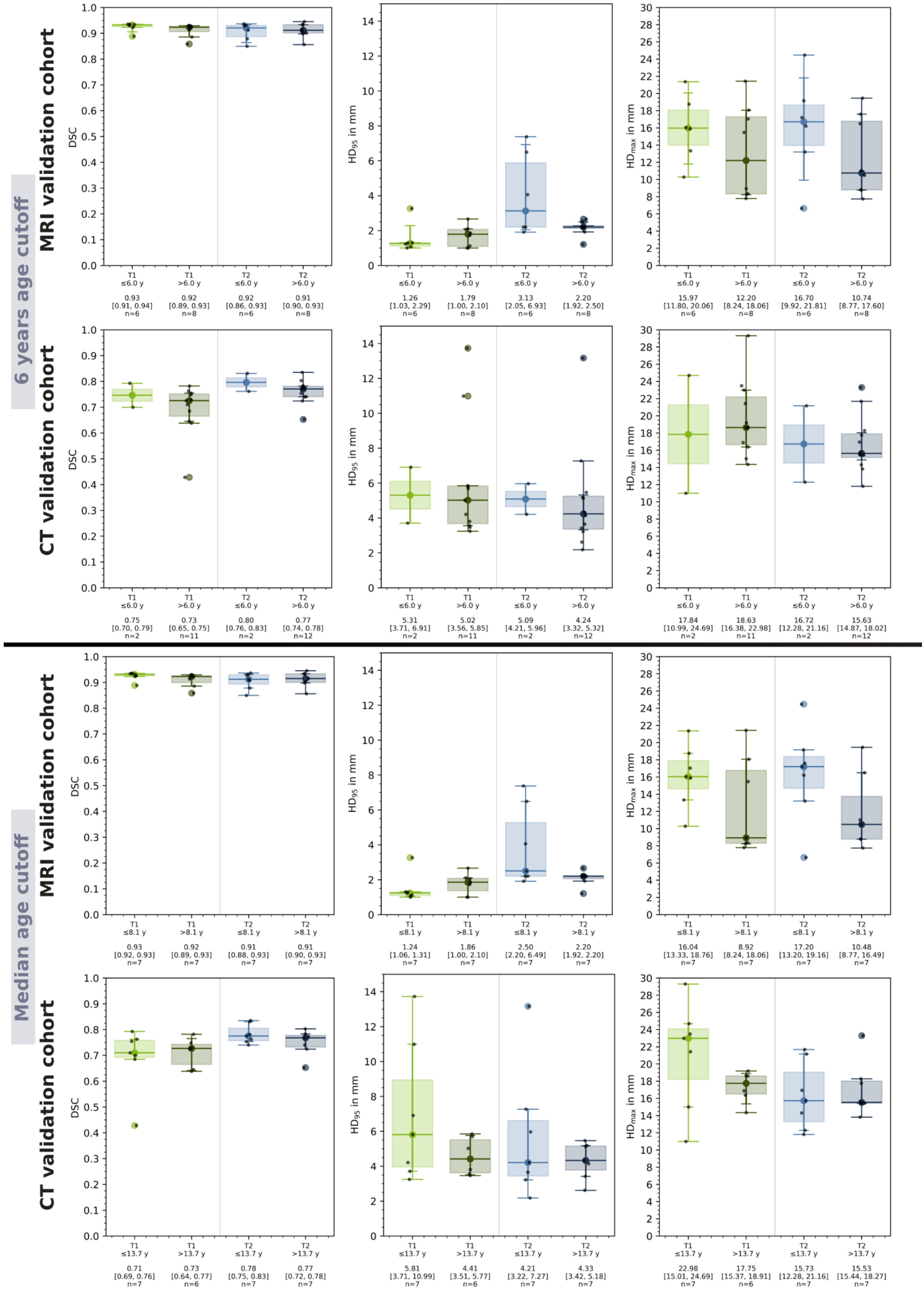

Figure S2. Age comparison with boxplots of the ventricle segmentation measures–Dice similarity coefficient (DSC) and $100^{th}/95^{th}$-percentile Hausdorff distance (HD_{max}/HD_{95})–for the T1 and T2 MRI weighting for the MR validation cohort and for the CT validation cohort after deformable registration with the brainstem as a focus region of interest and “mutual information” as the similarity measure. Above the thick black horizontal line: 6 years as age cutoff; below the thick black horizontal line: median age (of the respective validation cohort) as age cutoff. Boxes represent the interquartile range (IQR,Q_1-Q_2) with the median as a horizontal line. Whiskers extend to $[L, U] = [Q_1 - 1.5 \times \mathrm{IQR}, Q_3 + 1.5 \times \mathrm{IQR}]$; more extreme values are plotted as outliers $O = \{x_i : x_i < L \text{ or } x_i > U\}$. Inner vertical bars indicate bootstrapped 95% confidence intervals (CI) of the median. Due to the small number of cases per age group, the raw data for each case are shown as black scatter points. Where differences were statistically significant according to the Mann–Whitney U test, significance was indicated in the boxplot as $p < 0.05$.

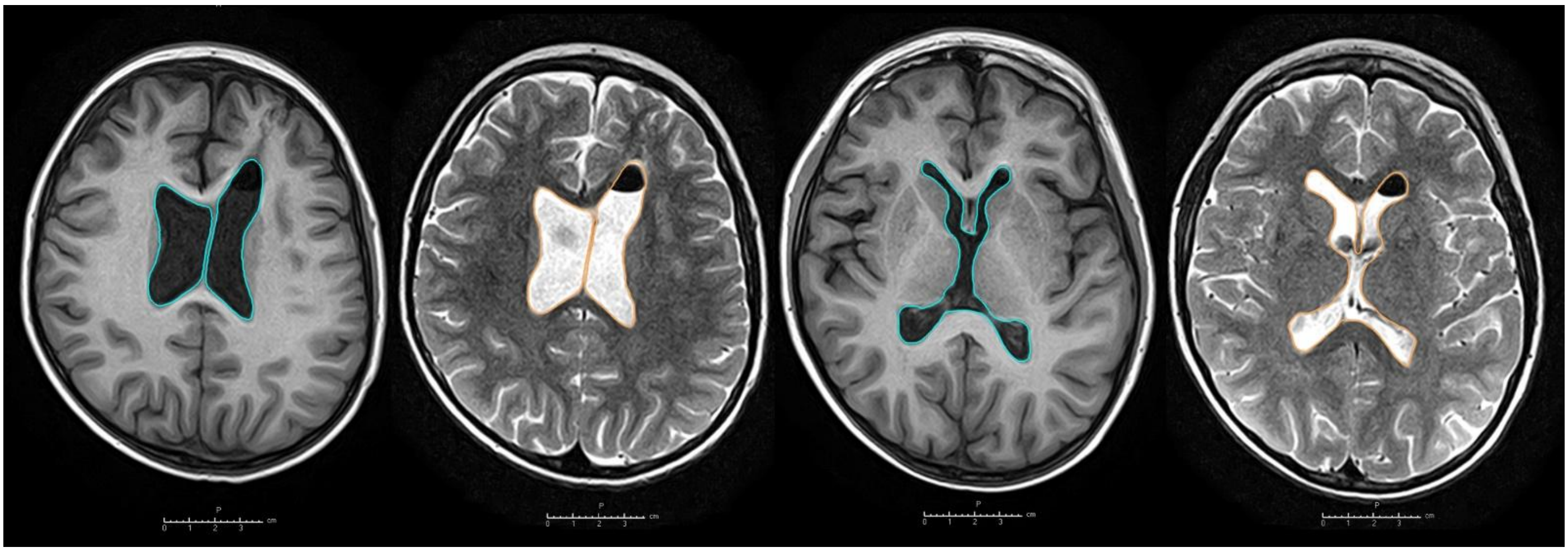

Figure S3. Deep learning-based lateral ventricles on T1- and T2-weighted MRI, shown exemplarily in two transverse slices each.

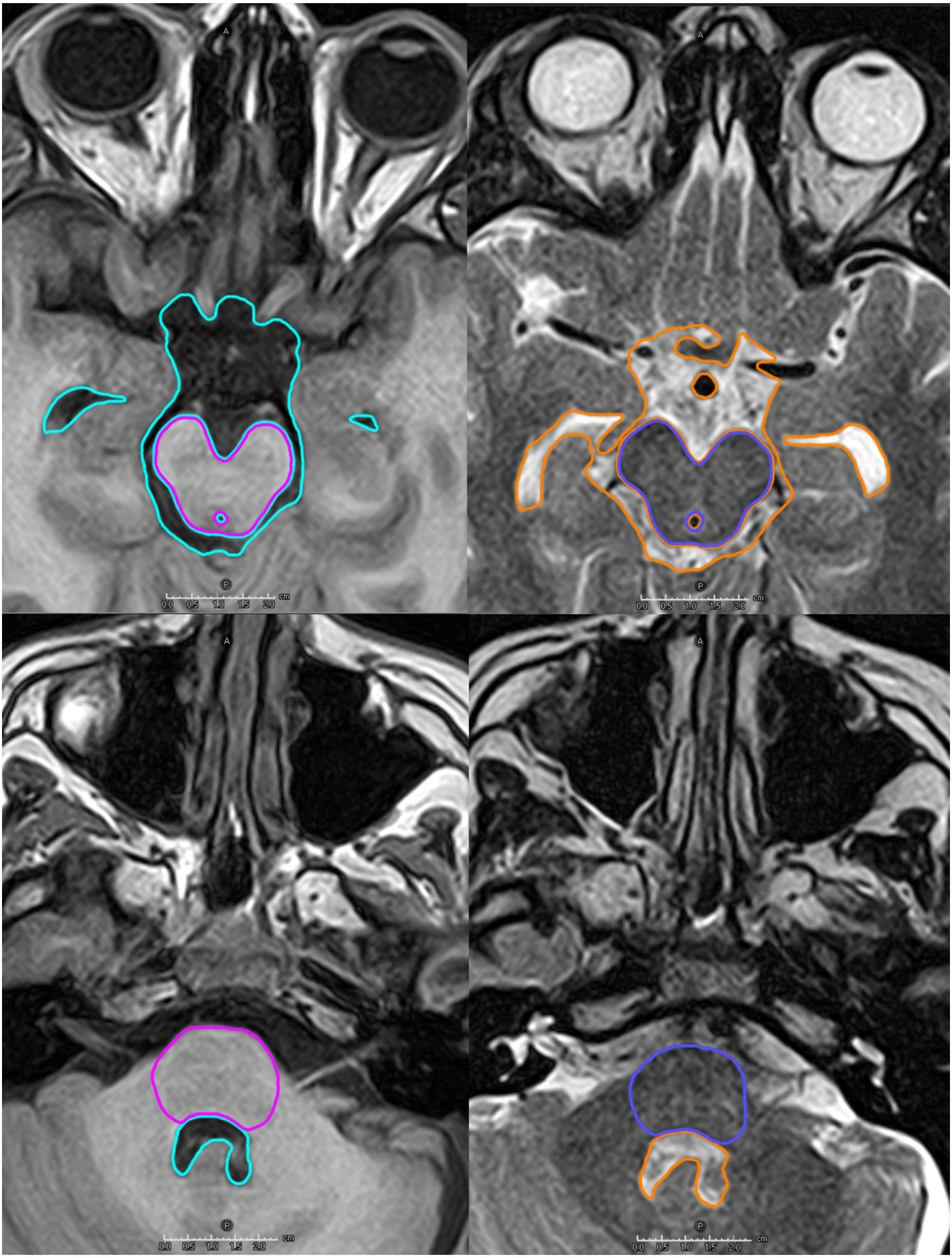

Figure S4. Deep learning-based ventricular system and brainstem on T1- and T2-weighted MRI (turquoise/orange: ventricular system; pink/violet: brainstem), shown exemplarily in two transverse slices each.

Supplementary material A

This section presents further details on the nnU-Net framework: the Dice similarity coefficient values from training, the python packages and its versions as well as the NIfTI conversion stats and the model weights. The inference scripts and further software requirements can be accessed at https://github.com/TIO-IKIM/Ventricle-Segmentation-Model. The trained model weights are available from the authors upon request.

Python packages:

- For DICOM to NIfTI conversion
- For nnU-Net training/inference
- For frontend/web GUI for inference viewer and case selection
- For converting nnU-Net predictions (NIfTI) to TPS-importable DICOM-SEG files

pydicom~=2.3.1[1]
dicompyler_core==0.5.5[2]
SimpleITK==2.2.1[3]
monai~=1.1.0[4]
nibabel~=5.0.0
nnunetv2==2.3.1[5]
numpy~=1.23.5
PyYAML==6.0
torch==1.13.1[6]
scipy~=1.10.0[7]
matplotlib~=3.7.0
tqdm~=4.64.1
torchmetrics==0.11.4
streamlit~=1.17.0
opencv-python~=4.7.0.68
pydicom_seg==0.4.1
rt_utils==1.2.5
pre-commit==3.0.3
DateTime~=5.0

[1] pydicom. GitHub - pydicom/pydicom at v2.3.1. GitHub n.d. https://github.com/pydicom/pydicom/tree/v2.3.1 (accessed September 28, 2026).

[2] dicompyler. GitHub - dicompyler/dicompyler-core at v0.5.5. GitHub n.d. https://github.com/dicompyler/dicompyler-core/tree/v0.5.5 (accessed September 28, 2026).

[3] Beare R., Lowekamp B., Yaniv Z. Image Segmentation, Registration and Characterization in R with SimpleITK. Journal of Statistical Software 2018;86:1–35. https://doi.org/10.18637/jss.v086.i08.;
Yaniv Z., Lowekamp B.C., Johnson H.J., Beare R. SimpleITK Image-Analysis Notebooks: a Collaborative Environment for Education and Reproducible Research. J Digit Imaging 2018;31:290–303. https://doi.org/10.1007/s10278-017-0037-8.;
Lowekamp B.C., Chen D.T., Ibanez L., Blezek D. The Design of SimpleITK. Front Neuroinform 2013;7. https://doi.org/10.3389/fninf.2013.00045.

[4] Cardoso M.J., Li W., Brown R., Ma N., Kerfoot E., Wang Y., et al. MONAI: An open-source framework for deep learning in healthcare 2022. https://doi.org/10.48550/arXiv.2211.02701.

[5] Isensee F., Jaeger P.F., Kohl S.A.A., Petersen J., Maier-Hein K.H. nnU-Net: a self-configuring method for deep learning-based biomedical image segmentation. Nat Methods 2021;18:203–11. https://doi.org/10.1038/s41592-020-01008-z.

[6] Ansel, J., Yang, E., He, H., Gimelshein, N., Jain, A., Voznesensky, M.,et al. (2024). PyTorch 2: Faster Machine Learning Through Dynamic Python Bytecode Transformation and Graph Compilation [Conference paper]. 29th ACM International Conference on Architectural Support for Programming Languages and Operating Systems, Volume 2 (ASPLOS '24). https://doi.org/10.1145/3620665.3640366

[7] Virtanen P., Gommers R., Oliphant T.E., Haberland M., Reddy T., Cournapeau D., et al. SciPy 1.0: fundamental algorithms for scientific computing in Python. Nat Methods 2020;17:261–72. https://doi.org/10.1038/s41592-019-0686-2.

Supplementary material B

This section presents further details on the application of the models in clinical practice and its use in retrospective and prospective cases. To assess voxel-discretization sensitivity, segmentation measures were recomputed across multiple grid resolutions of the 3D binary masks. Pairwise differences between grid resolutions were assessed using paired Wilcoxon signed-rank tests with Holm correction for multiple comparisons. Paired two one-sided tests (TOST) were additionally performed to assess equivalence within predefined equivalence bounds. An age subgroup analysis using Mann–Whitney U tests was considered exploratory.

Workflow for the clinical implementation of deep-learning-based segmentation:
Data exchange is managed through a hospital-internal web API that hosts the model and processes authenticated HTTP requests between the two involved institutions. Per-patient MR DICOM files are exported from RS to an internal network share, from which a Python client sends an authenticated request to a university hospital server. This server verifies credentials and DICOM tags, confirming the MRI sequence. For clinical implementation, the steps after the DICOM export are:

1. Generation of MR-based brainstem and VS contours on T1wIs and T2wIs via the DL model.
2. Storage of DL segmentations as two DICOM Radiotherapy Structure Sets objects associated with the corresponding MRI series information.
3. Import of DICOM structure set file with DL-generated structures into RS following consistency verification.
4. Mapping of the DL structures from the MRI to the planning CT (pCT) within RS:
    - 4.1 Selection of a registration method for each DL structure: rigid or deformable (with the DL brainstem used as a focus structure) registration.
    - 4.2 Verification of registration accuracy in RS by visual checks (multiplanar overlays) and anatomical landmarks when using rigid registration, and additionally by a heat map, warped grids, and vector arrows for observing the applied deformation vector field after deformable registration.
    - 4.3 Mapping of the contours.

Evaluation measures:
Quantitative contour-agreement was performed using a custom MATLAB R2024b[8] (MathWorks, Natick, MA, USA) script. Contour data extracted from DICOM RT Structure Set files were rasterized to a reference grid with the native CT/MRI spacing, yielding binary masks for voxel-wise comparison. Segmentation accuracy was quantified using the volumetric Dice similarity coefficient (DSC)[9] on the full 3D masks:

$$\mathrm{DSC} = \frac{2|\mathrm{A} \cap \mathrm{B}|}{|\mathrm{A}| + |\mathrm{B}|} \qquad \textit{Eq. (B.1),}$$

where A and B denote the predicted (DL) and ground truth/reference (manual) segmentation voxel sets, respectively, and the bidirectional Hausdorff distance (HD_{max}), which quantifies the maximum boundary deviation between two segmentations:

$$\mathrm{HD}(\partial A, \partial B) = \max\left\{\sup_{a\in\partial A}\inf_{b\in\partial B} d\,(a,b), \sup_{b\in\partial B}\inf_{a\in\partial A} d\,(a,b)\right\} \qquad \textit{Eq. (B.2),}$$

where $\sup_{a\in\partial A}\inf_{b\in\partial B} d\,(a,b)$ is the maximum, over all surface voxels $a \in \partial A$, of the minimum Euclidean distance $d(a,b)$ to any surface voxel $b \in \partial B$. To provide a more robust assessment, the 95th-percentile Hausdorff distance (HD_{95}) was also reported: $\mathrm{HD}_{95}(\partial A, \partial B) = \max\{P_{95}(\{\, d(a, \partial B) \mid a \in \partial A\,\}), P_{95}(\{\, d(b, \partial A) \mid b \in \partial B\,\})\}$.

For each quantitative measure, 95% confidence intervals (CIs) for the median were obtained using the percentile bootstrap method with 20,000 resamples and α =0.05.

[8] The MathWorks Inc. MATLAB version: 24.2.0.2863752 (R2024b) Update 5. 2022
[9] dice - Sørensen-Dice similarity coefficient for image segmentation - MATLAB n.d. https://www.mathworks.com/help/images/ref/dice.html (accessed April 22, 2026).

Image geometry and voxel volume:

The voxel geometry was derived from the DICOM metadata returned by dicomreadVolume. In-plane spacings were taken as $d_x =$ **Spatial.PixelSpacings(2)** and $d_y =$ **Spatial.PixelSpacings(1)**. Through-plane slice centers were obtained from the patient-coordinate z positions $\{z_i\} =$ **Spatial.PatientPositions(:,3)**. To be robust to small non-uniformities and to descending acquisitions, the nominal slice spacing was defined as $d_z = \text{median}(\Delta\, z_i)$ and we used $|d_z|$ in subsequent calculations. World limits were defined at voxel edges (not centers):

$$Z_{\text{limits}} = \left[\min(\{z_i\}) - \frac{|d_z|}{2}, \max(\{z_i\}) + \frac{|d_z|}{2}\right] \quad \textit{Eq. (B.3).}$$

Analogously, using the center of the first pixel
(x_0, y_0) =Spatial.PatientPositions(1,1:2) and the image size $(n_{\text{cols}}, n_{\text{rows}})$, the limits were set to

$$X_{\text{limits}} = \left[x_0 - \frac{|d_x|}{2},\ x_0 + (n_{\text{cols}} - \tfrac{1}{2})d_x\right] \quad \textit{Eq. (B.4),}$$

$$Y_{\text{limits}} = \left[y_0 - \frac{|d_y|}{2},\ y_0 + (n_{\text{rows}} - \tfrac{1}{2})d_y\right] \quad \textit{Eq. (B.5).}$$

These edge-aligned limits were passed to imref3d($[n_{\text{cols}}, n_{\text{rows}}, n_{\text{slices}}]$, X_{limits}, Y_{limits}, Z_{limits}), avoiding the center-to-center bias $(n-1)/\,n$. The voxel volume was then computed as $V_{\text{voxel}} = |d_x d_y d_z|$. This convention yields correct physical extents irrespective of slice order and provides numerically stable volumes and distances.

Equivalence testing of resampled MRI grids using Two One-Sided Paired t-Tests (TOST):

To assess whether metric values obtained on resampled grids were equivalent to those obtained on the reference grid (MRI grid), paired TOST were performed on the patient-wise differences $d = y - x$, where y denotes the value on the target grid and x the corresponding value on the reference grid. Resampling was performed on grids of 0.250, 0.500, 0.750, 1.000, and 2.000 mm. Performance-measure-specific equivalence margins were predefined as
Δ = 0.1 for DSC, Δ = 1.0 mm for HD_{95}, and Δ = 1.5 mm for HD_{max}. For each comparison, the null hypotheses $H_{01}: \mu_d \leq -\Delta$ and $H_{02}: \mu_d \geq \Delta$ were tested against the alternatives $H_{A1}: \mu_d > -\Delta$ and $H_{A2}: \mu_d < \Delta$, respectively, using one-sided paired t-tests at significance level α = 0.05. Equivalence was concluded only if both one-sided tests were significant, which is equivalent to the corresponding 90% confidence interval (1-2α = 0.9) of the mean paired difference lying entirely within the bounds $[-\Delta, \Delta]$, e.g., [−0.5,+0.5] mm for HD_{95}.
